# Enhancement of the BG-sentinel trap with varying number of mice for field sampling of male and female *Aedes albopictus* mosquitoes

**DOI:** 10.1186/s13071-016-1801-1

**Published:** 2016-09-22

**Authors:** Gilbert Le Goff, David Damiens, Laurent Payet, Abdoul-Hamid Ruttee, Frédéric Jean, Cyrille Lebon, Jean-Sébastien Dehecq, Louis-Clément Gouagna

**Affiliations:** 1 Institut de Recherche pour le Développement (IRD), UMR MIVEGEC (CNRS/IRD/UM), Maladies Infectieuses et Vecteurs, Ecologie, Génétique, Evolution et Contrôle, Montpellier, France; 2 IRD Réunion/GIP CYROI (Recherche Santé Bio-innovation), Sainte Clotilde, Reunion Island France; 3 Service de lutte anti vectorielle, Agence Régionale de Santé-Océan Indien (ARS-OI), Saint-Denis, Reunion Island France

**Keywords:** Arbovirus vector, Trapping mosquito, Sterile insect technique

## Abstract

**Background:**

Trapping male mosquitoes in the field is essential for the development of area-wide vector control programs with a sterile insect technique (SIT) component. To determine the optimal temporal and spatial release strategy, an estimation of the wild population density and its temporal dynamics is essential. Among the traps available for such data collection, the BG-Sentinel trap developed by the Biogents company uses a combination of visual cues, convection currents and olfactory signals. Although in numerous cases, this trap has shown high efficiency in sampling *Aedes albopictus*, in some cases low capture rates of *Ae. albopictus* males were recorded for the BG-sentinel mosquito trap baited with synthetic attractants.

**Methods:**

The effects of modifying the BG-sentinel trap (by adding one mouse, two or three live mice to the trap) on the efficiency of trapping *Ae. albopictus* males and females was tested. The experiment was carried out in three distinct areas on La Réunion that have been selected for pilot field testing of the release of sterile male *Ae. albopictus* mosquitoes. The effect of four types of attractant (including the generic BG-Lure, one mouse or two to three mice) in baited BGS traps was tested with a Latin square design in order to control for the variability of different sampling positions and dates.

**Results:**

At the three studied sites, the number of *Ae. albopictus* adults caught and the proportion of males per trap consistently increased with the number of mice present in the trap.

**Conclusion:**

The results from this study suggest that some new attractants derived from, or similar to, mouse odors could be developed and tested in combination with other existing attractive components, such as CO_2_ and heat, in order to provide a reliable estimation method for *Ae. albopictus* adult male abundance in the wild.

**Electronic supplementary material:**

The online version of this article (doi:10.1186/s13071-016-1801-1) contains supplementary material, which is available to authorized users.

## Background

Monitoring vector populations in endemic areas is essential to understand their ecology, to estimate their abundance and finally for the planning of vector control measures. Since female mosquitoes are responsible for disease transmission, most of the commonly used traps have been designed to attract female mosquitoes [1, 2]. Although the monitoring of males is also essential for area-wide vector control programs including the sterile insect technique (SIT), trapping male mosquitoes in the field has been challenging. The SIT is a biological control method used to control insect pests by releasing a large number of sterile males into the wild population. These will compete with wild males to mate with females in the field and thereby reduce the fertility of the target population [3, 4]. The success of SIT relies mainly on a convenient release strategy that takes into account the number of sterile males released, the frequency of the releases and the reproductive quality of these males [5]. To determine the optimal temporal and spatial release strategy, an estimation of the wild population density and its temporal dynamics is essential [6]. The population density may be estimated by mark-release-recapture (MRR) experiments [1, 7, 8]. This same technique has been used for estimating the size of wild *Aedes* species populations [9–13], and has also been used to determine the quality of the mass reared and sterilized males by estimating their survival and their dispersal ability in the field after release [14–17]. Mark-release-recapture techniques consist of the release of mass produced insects usually marked with fluorescent dyes, and the daily recapture over several days post release using numerous traps placed at different distances from the release point [1, 7, 8]. The ability to conveniently apply this MRR technique for estimating the population size of male *Aedes albopictus* relies heavily on the availability of efficient male trapping methods and sampling tools.

*Aedes albopictus* is the main vector (and most likely sole vector) of dengue and chikungunya on La Réunion Island, and has been implicated in the massive epidemic that occurred in the Southwest Indian Ocean islands from 2004 to 2007 [18]. Recently, comprehensive studies on the biology and ecology of *Ae. albopictus* have led to establish the capacity to implement SIT programmes on the island. We have improved mass rearing methods, and detailed baseline entomological data on all biological parameters of adult mosquitoes needed to establish the specific biological and behavioural determinants that contribute to sexual competitiveness of sterile males, in laboratory [19] and semi-field [20, 21] experiments. In order to establish baseline spatial and temporal information about the target population for future pilot testing, two potential pilot sites were chosen in which intensive surveillance of the population of *Ae. albopictus* was undertaken between 2013 and 2015 using oviposition traps and BG-sentinel (BGS) traps (Le Goff et al. unpublished data). The BGS traps developed by the Biogents (Regensburg,, Germany) in 2006 use a combination of visual cues (contrasting black and white colours), convection currents (similar to those generated by humans) and a BG-Lure® as an olfactory signal [22]. The BG-Lure® consists of synthetic compounds such as lactic acid, ammonia and caproic acid (hexanoic acid) that mimic the odour of human skin [23]. Although this trap was initially developed to sample *Ae. aegypti,* it is also efficient in collecting *Ae. albopictus* [24–30] and a large range of other mosquito species [31].

During early field observations on La Réunion Island [32], and field reports elsewhere focusing on the use of attractants for collecting *Ae. albopictus* [33], the capture rates using BGS traps with a synthetic attractant were low and were numerically dominated by *Ae. albopictus* females. Results from more recent field investigations on La Réunion Island [13, 15] showed that mice-baited BGS traps were considerably more attractive to *Ae. albopictus* than traps baited with lure alone. However, none of these studies was specifically designed to calibrate the standard BGS trap for the collection of *Ae. albopictus* males*,* and no efficacy field trials have ever been designed previously to purposively compare and standardise the response of wild male and female *Ae. albopictus* to BGS traps baited with a varying number of mice.

The purpose of the present study was to address the hypothesis that the use of mice as baits can enhance the yield of BGS traps and increase sampling efficiency for catching *Ae. albopictus* males. Specifically, we aimed at determining the optimal number of mice required to enhance the sensitivity of BGS traps in sampling male *Ae. albopictus*. This was accomplished by comparing the BGS traps baited either with the generic BG-Lure, with one mouse, two or three mice in two different pilot field sites selected for the control of *Ae. albopictus* using sterile male releases and in one low mosquito density buffer zone that separates the two field sites. This information is relevant particularly in the context of SIT planning, implementation and evaluation.

## Methods

### Classic BGS trap

**Fig. 1:**
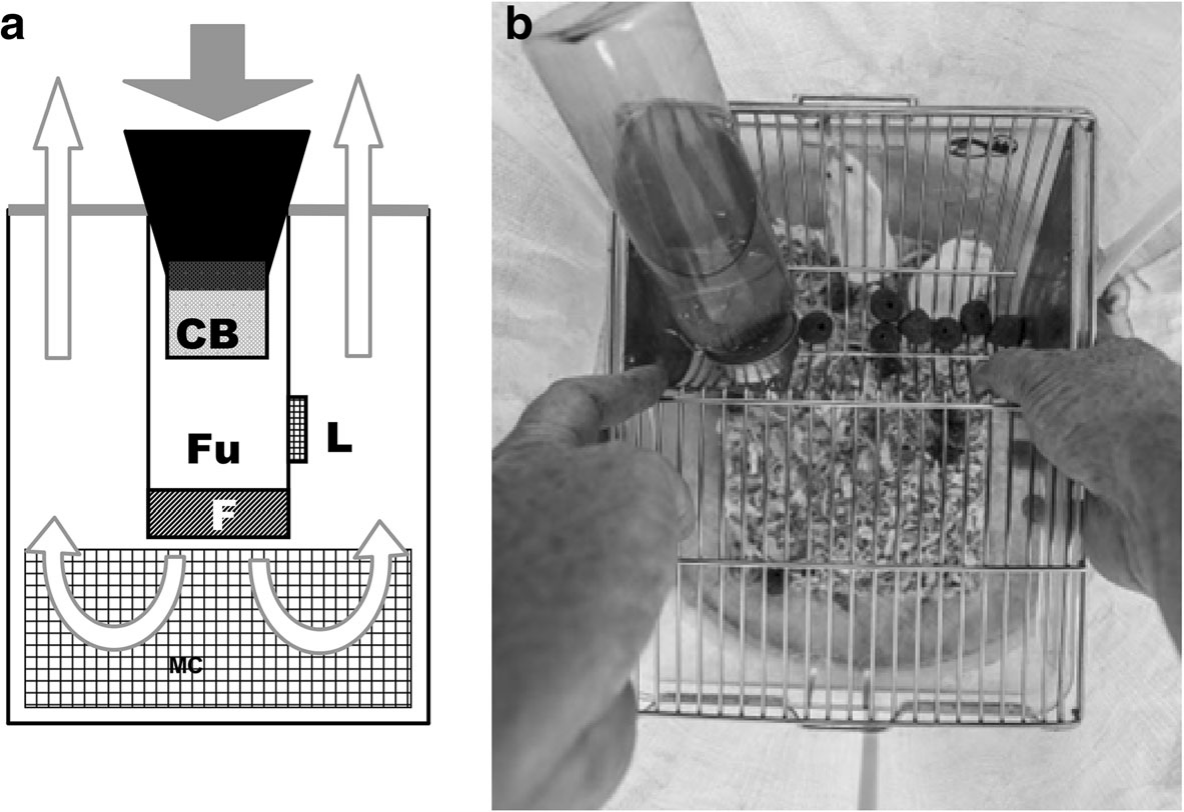
**a** BG-sentinel trap in vertical section in its classical form with the BG-Lure (*L*) and without the Mouse cage (*MC*), and in its modified form without the BG-Lure and with the Mouse cage (*MC*). The *white arrows* indicate the flow of air coming out from the trap and blowing out the BG-Lure odour or the mouse odour. The *grey arrow* indicates the flow of air sucking in the flying insects. **b** Mouse cage in the bottom of the BG trap. *Abbreviations*: *F* fan, *Fu* funnel, *CB* collection bag, *L* Lure, *MC* mouse cage

The BGS trap is a lightweight collapsible cylinder (40 cm high, 36 cm diameter), closed at its base and covered by a white net on the top (Fig. 1a). In the middle of the cylinder there is a cylindrical funnel (15 cm) with a small fan at the bottom powered by a 12 V/9 Ah rechargeable sealed lead acid battery (FIAMM-AGM Technology, Aubergenville, France). The fan blows air downward into the cylindrical funnel that is equipped with a fine mesh insect collection bag. The fan also blows air and the lure odour (placed on the outside of the funnel) upward and out through the netted cover.

### Mice-baited BGS trap

The classic BGS trap was modified to accommodate a cage containing 3 mice placed at its base (Fig. 1). The fan blew air upward and out through the netted cover disseminating also the mouse odour. Mice were placed in a clear rearing polycarbonate cage (265 × 205 × 140 mm) equipped with a stainless steel tray by which food and water were provided. Dog food pellets were used to feed the mice, while water was provided through a clear polycarbonate bibber with stainless steel sipper.

### Study areas

**Fig. 2:**
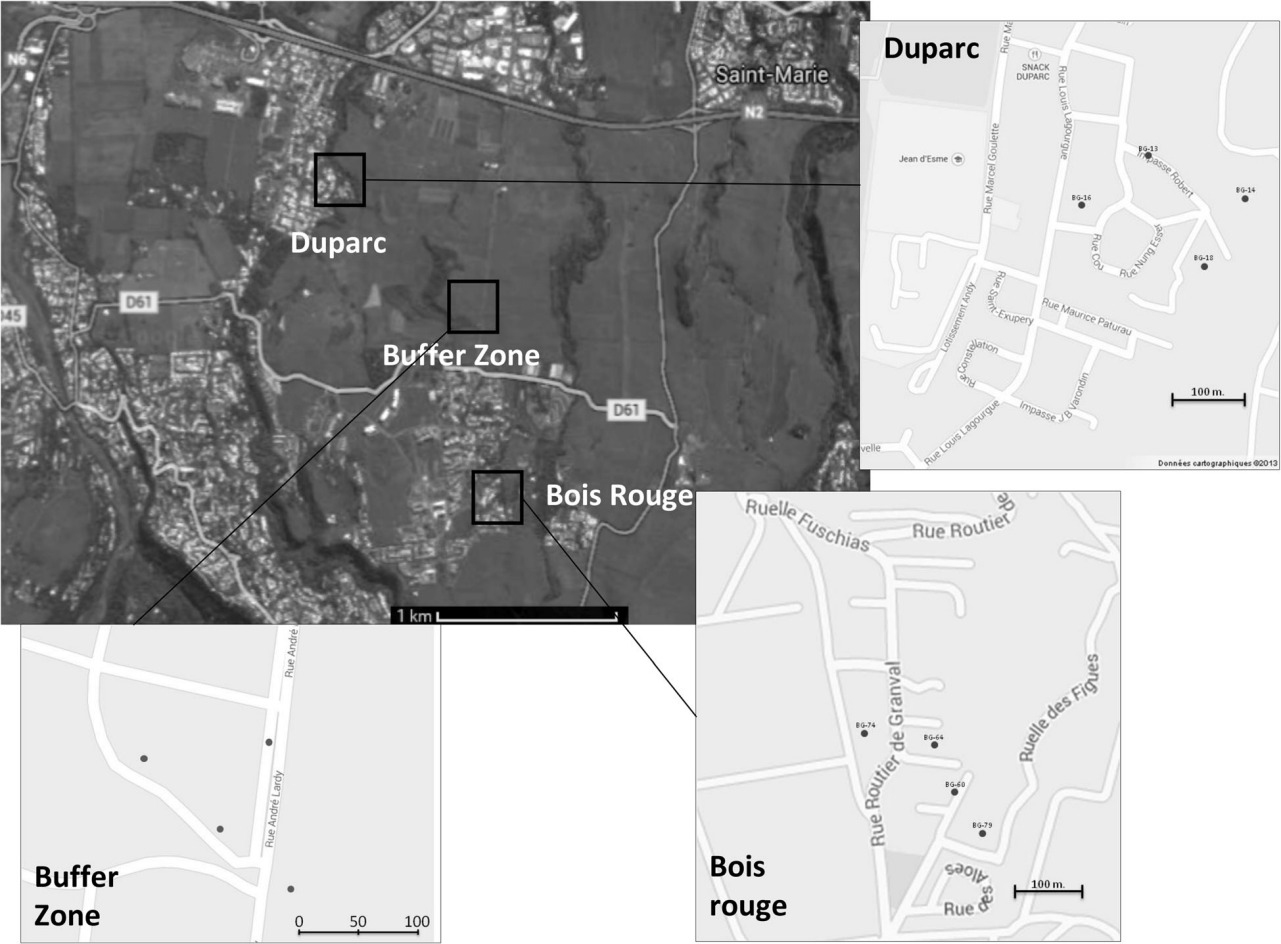
Localisation of the three collection areas: two urban pilot sites, Duparc and Bois Rouge, and the uninhabited buffer zone. Dots indicate the position of the four types of traps at each collection site

The experiment was carried out on La Réunion Island in three distinct areas (Fig. 2): two urban zones, 1 km away from each other, previously selected as pilot field sites for the control of *Ae. albopictus* using sterile male releases, and an uninhabited buffer zone. The urban pilot sites ‘Duparc’ and ‘Bois Rouge’ are located in the city of Sainte Marie within the northern district of La Réunion Island. Duparc is a 22 ha urban zone with 373 premises that cover 22 % of the area. Elevation of the site ranged from 50 to 80 m above sea level (asl). In the north, the site is isolated by an expressway linking Saint Denis to Saint Benoit, by La Mare Ravine in the east and by sugar cane fields to the west and south. The Bois rouge study site is a 24 ha area with an elevation ranging from 150 to 210 m asl and 262 premises that cover 21 % of the surface. The site is isolated by the presence of sugar cane fields to the north, east and south. The third site separates the two pilot sites providing a buffer zone. This buffer zone consists of a sugar cane field with shrubs and grass (Fig. 2), in which the population of mosquitoes has been shown to be very low or null.

### Mosquito sampling design

Four different attractants were tested: BGS traps with the BG-Lure and BGS traps with 1, 2 or 3 mice. While female and male mice coexist well with each other in 1 cage, the presence of 2 male mice in a cage can lead to fatal fights. For this reason, all males were placed either separately, 1 in a cage or together with 1 or 2 females. The mice used were between 8 and 12 weeks old. For each study area, 4 positions were determined, separated from each other by a minimum of 50 m and a maximum of 100 m (Fig. 2) to avoid interference between traps. To take into account the effect of different positions on the number of mosquitoes captured by one type of trap, each trap was rotated between each location every 24 h. Three replicates were performed between 20th and 24th of April, 4th and 8th of May and 18th and 22nd of May 2015 (i.e. during the week No. 17, 19 and 21, respectively), and one replicate consisted of one complete trapping cycle of 4 × 24-h trapping periods. Traps were placed on the ground in a shaded location. They were activated every day between 8:30 and 10:30 and mosquitoes were collected the day after at the same time. Batteries (12 V) were changed every day and a group of mice were left in the field for the first two consecutive days and were replaced by new mice for the last 2 days. The insect capture bags were collected daily and brought to the laboratory where field collected mosquito samples were individually identified using morphological characteristics. The number of *Ae. albopictus* adults and the male ratio (defined as the number of males caught divided by the total number of *Ae. albopictus* adults caught) were recorded for each collection.

### Statistics

For general observations, proportions of mosquitoes caught in traps according to the type of bait, the week of collection or the different collection sites were compared using the G-test [34]. We compared the observed counts of the numbers of observations in each category (type of baits, the week of collection or the different collection sites) with the expected counts, which we calculated here as the theoretical expectation if the same proportion of mosquitoes was caught in each trap (such as a 1:1:1:1 ratio for the type of trap or a 1:1:1 ratio for the 3 weeks of collection or the different collection sites). For the *post-hoc* test, we determined which categories were significantly different from their null hypothesis by testing each category *vs* the sum of all categories, with the Bonferroni correction.

The effect of the four types of attractant was tested while controlling for the variability of the four different positions (in this case the Latin square number) and the four different trapping periods. Here, the Latin square design was replicated three times. In our conditions, we kept the same row (Date) and column (Position) levels giving three identical squares for the three replicates that could be analysed through a General Linear Model (GLM) test in Minitab 16 [35]. The GLM procedure was performed using two response variables: the total number of *Ae. albopictus* adults collected in each trap and the male ratio within the caught samples; while fixed independent variables were the position (of the trap) and date and the covariate was the attractant (BG-Lure, 1, 2 or 3 mice). Multiple comparison procedures (Tukey’s HSD tests) were also performed to test significant differences in the number of caught mosquitoes among different traps. All analyses were run in Minitab statistical package.

## Results

### General observations

A total of 2336 adult mosquitoes were collected over the course of the study. Only three species were found among the captured mosquitoes: *Ae. albopictus* with 1862 adults (1055 females and 807 males), *Culex quinquefasciatus* with 473 adults captured (228 males and 245 females) and only 1 *Lutzia tigripes* female. In total, 902 (48 %), 495 (27 %), 309 (17 %) and 156 (8 %) *Ae. albopictus* were captured in the 3, 2 or 1 mouse and lure BG traps, respectively (G-test, *G *= 659.85, *df* = 3, *P* < 0.001). For *Cx. quinquefasciatus*, 209 (44 %), 84 (18 %), 118 (25 %) and 62 (13 %) adults were collected in the 3, 2 or 1 mouse and lure BGS traps, respectively (G-test, *G* = 100.05, *df* = 3, *P* < 0.001). The number of adults captured during the three 3 replicates were 789, 432 and 641 for *Ae. albopictus* (G-test, *G* = 106.92, *df* = 2, *P* < 0.001) and 208, 169 and 96 for *Cx. quinquefasciatus* for the week 1, 2 and 3, respectively (G-test, *G* = 43.50, *df* = 2, *P* < 0.001). For the whole duration of the experiment, 608, 1053 and 201 *Ae. albopictus* (G-test, *G* = 634.92, *df* = 2, *P* < 0.001) and 174, 233 and 66 *Cx. quinquefasciatus* (G-test, *G* = 101.35, *df* = 2, *P* < 0.001) were collected from the two urban environments in Bois Rouge, Duparc and the Buffer Zone, respectively. For *Ae. albopictus*, the proportions of adults caught during the course of the experiment in different sites were significantly different from the expected proportion (assuming the same proportions of mosquitoes were caught in each site) for Duparc and the Buffer Zone, but not for Bois Rouge.

### Relationship between the number of mice and the number of caught *Ae. albopictus* per trap

**Fig. 3:**
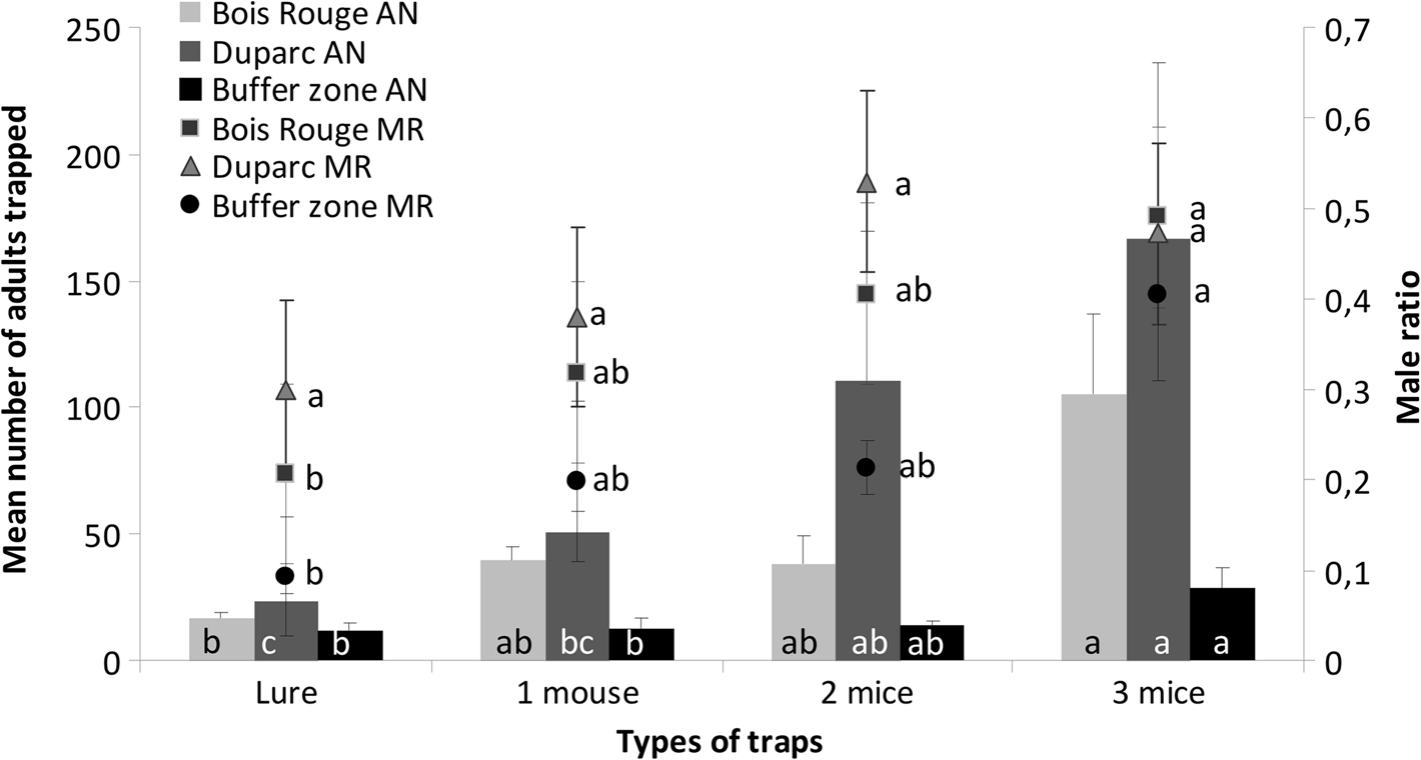
Mean number of adults (males and females) (bars) and mean male ratio (defined as the number of males caught divided by the total number of adults caught) (line) of those adults caught per week by traps with different number of mice as baits. Columns and points with the same letter indicates that the results are not significantly different (*P* > 0.05, Tukey’s HSD *post-hoc* test following a GLM procedure within each zone)

The number of adults (males and females) and the ratio of male mosquitoes to the total number of adults caught per day by traps with the different numbers of mice attractants are presented in Fig. 3. The number of adults caught varied significantly with the number of mice present in the trap in Bois Rouge (GLM, *F*_(3,34)_ = 6.37, *P* = 0.002), Duparc (GLM, *F*_(3,35)_ = 8.74, *P* < 0.0001) and in the low density buffer zone (GLM, *F*_(3,35)_ = 5.16, *P* = 0.005). At all three studied sites, BGS traps baited with three mice caught the greatest number of mosquitoes compared to traps with the BG-Lure and those traps with one mouse. The BGS traps containing two mice did not catch significantly more adult mosquitoes compared to all other traps, except in Duparc where it was significantly different from that recorded in the traps using the BG-Lure. In the low density zone (the buffer zone), where less than ten adults were caught during the four consecutive sampling days, BGS traps with the BG-Lure and BGS traps baited with one or two mice caught a similar number of adults, while BGS traps baited with three mice showed a significantly greater yield compared to the BGS traps using the BG-Lure, or 1 mouse (*P* < 0.05 in all cases).

### The effect of varying number of mice in trapping devices on the male ratio among caught individuals

At the Duparc site, no significant difference in the male ratio (defined as the number of males caught divided by the number of caught adults per trapping period) was seen among traps using different attractants (GLM, *F*_(3,36)_ = 0.50, *P* =0.687, Fig. 3). In contrast, the male ratio varied significantly with each different type of attractant used at the Bois Rouge site (GLM, *F*_(3,36)_ = 2.99, *P* = 0.044) and at the Buffer zone (GLM, *F*_(3,33)_ = 3.51, *P* < 0.025). Indeed, in these zones, the number of caught males/total adults per trap with the BG-Lure was significantly lower than that recorded for traps baited with three mice (Fig. 3), in which a significantly higher number of males per trapping period were caught than with traps baited with only one or two mice.

## Discussion

Consistent with previous investigations in which the dispersal [15] or the population size [13] of *Ae. albopictus* was evaluated in the field, the present results confirm that a mouse-baited BG-Sentinel trap provides an efficient tool for monitoring both *Ae. albopictus* males and females. The presence of three mice placed at the bottom of the trap significantly increased the number of adult mosquitoes caught, and the proportion of males caught.

The attraction of *Ae. albopictus* to mice seems logical since the species is generally distributed in rural and natural areas and feeds on humans, but also on numerous animal species including amphibians, reptiles, birds and mammals [36–39]. The use of ‘natural host odours’ such as human skin odours on worn socks has already been shown to increase *Ae. aegypti* collections in traps when compared to traps baited with the BG-Lure [40, 41].

Several reasons may explain the enhanced attractiveness of the trap with the increasing number of mice. Indeed, the ability of mosquitoes to locate a blood meal has already been described as a behavioural response to a complex blend of host parameters [42] such as host skin odours [43], excretion odours, breath volatiles [44], warm and moist convective currents or CO_2_ emission [45, 46]. These attractive components are probably increased with the number of mice.

Heat and moisture emanating from the mice-holding cage are expected to be an important factor influencing the level of attraction of mosquitoes to the traps. Indeed, males and host seeking females may respond to the quantity of body heat and moist convective currents as they make their final approach to a trap during the attraction stage. Although previous experiments have indicated that grouped animals may elicit higher body temperatures than do isolated animals, the effect of heat was not purposely tested in the present study [47].

Carbon dioxide is known to be the most effective mosquito attractant at long and medium distances [48, 49]. The presence of three mice will lead to a production of CO_2_ [50] three times higher than that of only one mouse and could explain the higher efficiency of the trap baited with three mice. In recent studies [33], significantly more *Ae. albopictus* were caught in CO_2_-baited traps (either in the form of dry ice or compressed gas) than in unbaited traps.

The presence of mice of different sex in the trap could have induced some behaviour that increased the odors in the given set up. Indeed, in the one-mouse cage, the mouse was usually a male, whereas for those containing two mice, one male and one female were typically used, and two females were placed with one male in cages with three mice. The presence of two males in the same cages was avoided to prevent fighting which could lead to deadly injuries. The presence of mixed sexes in the two and three mice traps could have changed the urinary volatiles composition for example, which could be very different according to the sex and endocrine status of the animals [51, 52].

In the current experiment, the litter impregnated with odours from mouse excretions (such as urine and faeces) have added to the odours and heat from the mouse itself to trigger increased mosquito attraction to traps [51, 53]. These odours that regulate the social behaviour in the mouse [54–56], could also be used by mosquitoes to detect a host as observed in a preliminary experiment by Le Goff (personal observation) who also saw increased BGS trap efficiency when a small container with 1 week old litter was placed in the BGS trap in lieu of mice. However, in the present study this effect is probably limited since cages in the traps were changed every 2 days.

In several species of *Aedes* mosquitoes, males may assemble in the vicinity of the host presumably to intercept females coming to feed [57, 58]. Whilst a lower proportion of males were attracted to traps baited with the synthetic lure and one mouse, it is unclear whether the increased ratio of males to females in traps with two or three mice involved olfactory cues emanating from the mice. More in-depth studies are needed in order to determine the role of host odours on male *Ae. albopictus* behaviour. Nevertheless, it is plausible that the attraction of males may be accrued only by the perception of the higher number of females, that was related to the increasing number of mice per trap.

## Conclusions

The current study demonstrated that the use of BGS traps baited with two or three mice could increase their efficiency to catch and sample more male mosquitoes than BGS traps with the BG-Lure alone. Moreover, in areas with low mosquito densities such as in the buffer zone, the use of BGS traps baited with three mice will significantly enhance our ability to detect the presence mosquitoes compared to all other set ups.

Large scale mosquito monitoring using baited traps could be problematic due to the additional necessity of rearing mice, which requires space, intensive labour and is time consuming. In addition, the licensing of activities involving animal rearing and animal welfare legislations could be constraining. The present study suggests that new attractants derived from mice odours could be developed and tested, possibly in association with a CO_2_ source or with other existing attractive components [59] in an attempt to both enhance the performance of commonly used trapping tools and to increase adult *Ae. albopictus* male sampling success. The use of convenient male trapping systems in the SIT context is needed both to establish baseline spatial and temporal information about the target population size, and to gain insights about on-site dispersal and mating activity of released sterile males. This information could be useful for making informed decisions regarding the scale of the release program and assess the level of success of such interventions.

## Additional file

Additional file 1: Total data set of female and male *Aedes albopictus* recapture according to the replicate, the site, the location and the bait of the trap. (XLSX 22 kb)

## Abbreviations

SIT: Sterile insect technique
BG: Biogent
BGS: Biogent-sentinel trap
MRR: Mark-release-recapture
FIAMM-AGM: Technology, (‘Fabbrica Italiana Accumulatori Motocarri Montecchio’-absorbent glass material technology)
ASL: Above sea level
GLM: General linear model
